# Long-term efficacy of crizotinib in a metastatic papillary renal carcinoma with *MET* amplification: a case report and literature review

**DOI:** 10.1186/s12885-018-5049-3

**Published:** 2018-11-22

**Authors:** Philippe Rochigneux, Jeanne Thomassin-Piana, Sophy Laibe, Serge Brunelle, Naji Salem, Bernard Escudier, Gilles Vassal, Gwenaelle Gravis

**Affiliations:** 1 Department of Medical Oncology, Institut Paoli-Calmettes, 232 Bd de Sainte-Marguerite, 13009 Marseille, France; 2 UCLA David Geffen School of Medicine, Los Angeles, USA; 3 Department of Pathology, Institut Paoli-Calmettes, Marseille, France; 4 Department of Cytogenetics and Molecular Genetics, Institut Paoli-Calmettes, Marseille, France; 5 Department of Radiology, Institut Paoli-Calmettes, Marseille, France; 6 Department of Radiotherapy, Institut Paoli-Calmettes, Marseille, France; 7 Department of Medical Oncology, Gustave Roussy Cancer Center, Villejuif, France; 8 Direction of Clinical Research, Gustave Roussy Cancer Center, Villejuif, France

**Keywords:** Papillary renal cell carcinoma, MET, MET inhibitor, Crizotinib, Radiotherapy

## Abstract

**Background:**

Papillary renal cell carcinoma (pRCC) is the 2nd most frequent histological type of kidney cancer and accounts for approximately 15% of all renal cell carcinoma. It has a poorer prognosis than clear cell RCC (ccRCC) with a lack of standard treatments.

**Case presentation:**

We report the case of a 51 year old man with a metastatic pRCC (hepatic dome and left colonic peritoneal carcinomatosis) progressive after sunitinib, with a *MET* amplification. The patient was enrolled in the UNICANCER-sponsored AcSé crizotinib trial (NCT02034981), designed to give an access to crizotinib for patients with tumors harboring a genomic alteration on one of the biological targets of the drug. With 2nd line crizotinib (250 mg twice/day), the patient had a very good tolerance, a partial response in the target lesions using RECIST 1.1, and a 19 months’ clinical efficacy.

**Conclusions:**

In metastatic pRCC with a MET amplification, crizotinib maybe a potential met-inhibitory therapeutic option.

## Background

Papillary renal cell carcinoma (pRCC) is the 2nd most frequent histological type of kidney cancer and accounts for approximately 15% of all renal cell carcinoma (RCC). The classifications distinguish two types of pRCC: type 1 (more indolent, with basophilic cells) and type 2 (more aggressive, with eosinophilic cells). The prognosis of metastatic pRCC (mpRCC) is worse than in clear cell RCC (ccRCC), with a median PFS around 6 months and a median OS around 12 months, versus 10.5 months and 15.7 months respectively for ccRCC [1, 2]. For mpRCC, sunitinib or mTor inhibitors are proposed by NCCN Guidelines, but the inclusion in a clinical trial is the preferred option [3]. MET protein is a heterodimer transmembrane Tyr-Kinase receptor, with one known ligand (Hepatocyte Growth Factor) and a signaling via MAP-Kinase pathway, leading to increase proliferative functions (invasion, aggressiveness, angiogenesis) [4]. Anomalies of *MET* pathway have been described in several tumors (NSCLC, gastric, ovaries), and particularly in pRCC where 75% of hereditary pRCC have activating mutations in the Tyr/Kin domain [4, 5]. Here we present the case of a patient with a *MET*-amplified mpRCC successfully treated during 19 months with the MET-inhibitor crizotinib within the French AcSé- crizotinib program [6].

## Case presentation

**Fig. 1:**
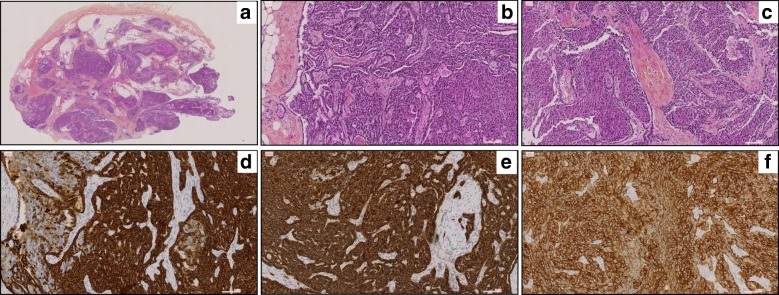
Microphotographs showing a morphology of papillary renal cell carcinoma, with papillary and foamy macrophages (**a**) (**b**) (**c**). Tumoral cells expressed CK7 (**d**), Racemase/P504S (**e**), and C-MET +++ (**f**)

We describe a patient born in 1961, a manager in the food processing industry, diagnosed with a right renal mass of 50 mm in February 2012, leading to a partial right nephrectomy. Histopathological examination revealed a tubulo-papillary renal cell carcinoma, type 1, grade 2 of Fuhrman, 20% of necrosis, without vascular embolus or peri-renal infiltration, R0, pT1bNx, vimentin +, CD 10+. In September 2013, an abdominal computed tomography scan (CT scan) showed a nodular peritoneal infiltration (right peri-colic), with a sub-cutaneous nodule of the right flank, a mild intraperitoneal effusion in the Douglas pouch, and a pre-rectal nodule. After a control CT scan, confirming the stability of the lesions, surgery was performed. The pathological findings (Fig. 1) confirmed the morphology of papillary renal cell carcinoma, with papillary and foamy macrophages (A, B, C) and a positivity of CK7 (D), Racemase/P504S (E) with immunohistochemistry (IHC).

The patient was referred to our Comprehensive Cancer Center in April 2014, with a post-operative abdominal mass. His medical history included cigarette smoking (20 packs/year), thyroidectomy (supplementation), non-insulin-dependent mellitus diabetes (metformin) and hypertension (amlodipine, valsartan). Clinically, he weighed 78 kg for 1.80 m, Karnosky performance scale was 80% and physical examination revealed induration of the abdominal scar. The laboratory tests were normal (Hb = 14.6 g/dl, Neutrophil = 2.3 10^9^/L, Platelets = 326 G/l, absence of hypercalcemia) and the patient was classified in the favorable-risk group according to Heng criteria. Repeat CT scan revealed peritoneal carcinomatosis.

**Fig. 2:**
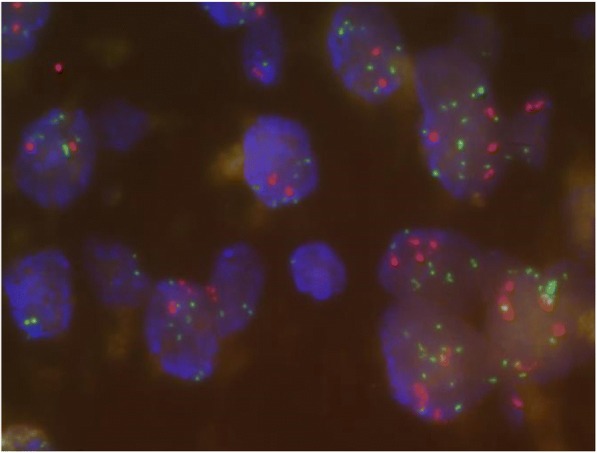
Fluorescence in situ hybridization (FISH) of *MET*, realized with a MET/CEN7 Probe (ZytoLight**®** SPEC MET/CEN7 Dual Color Probe, ZytoVision**®**): *MET* in green, and centromere of chromosome 7 in red). The result showed a *MET* amplification (*MET* > 6 copies per cell, and a ratio *MET*/CEN7 > 2)

After a multidisciplinary board, a treatment by anti-angiogenic sunitinib started in May 2014. Toxicity was mild with grade 1 nausea, grade 2 oesophagitis, and abdominal ascites requiring three ascitic drains. The increase of the peritoneal carcinomatosis was observed on the follow-up CT scan. For this progression, complementary IHC were performed (Fig. 1f), revealing a cMET 100% (cytoplasmic antibody staining, Roche Diagnostic clone SP44, catalog number 790–4430), a cMET score 3+ (based on number of positive cells and staining intensity), with ROS -, ALK – and no loss of expression of PTEN. This cMET positivity was confirmed by Fluorescence In Situ Hybridization (FISH) of *MET* (Fig. 2) realized with a MET/CEN7 Probe (ZytoLight**®** SPEC MET/CEN7 Dual Color Probe, ZytoVision**®**: *MET* in green, centromere of chromosome 7 in red). The result showed a *MET* amplification (80% of positive cells, *MET* > 6 copies per cell, ratio *MET*/CEN7 > 2). The absence of chromosome 7 and 12 polysomy was verified in a complementary FISH.

**Fig. 3:**
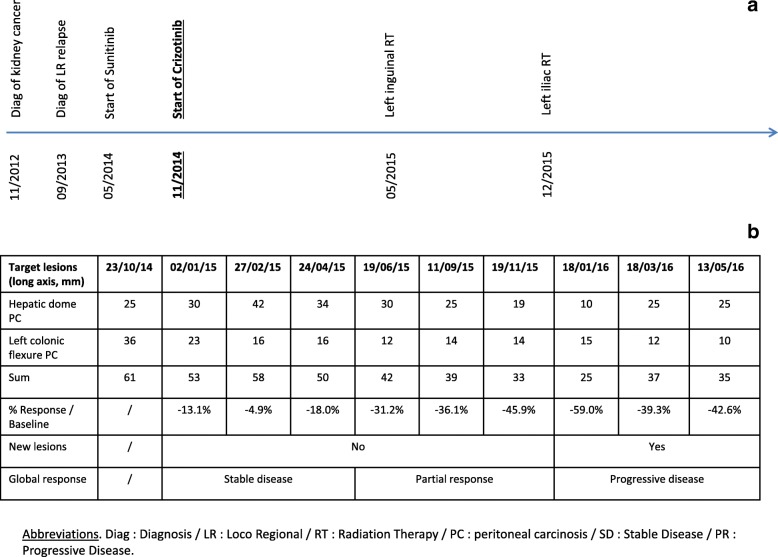
Chronological evolution of target lesions assessed by CT scan, with the timeline in Panel (**a**) and the RECIST 1.1 response evaluation in Panel (**b**)

After a specific molecular tumor board, the patient was enrolled in the UNICANCER-sponsored AcSé crizotinib trial (NCT02034981) allowing patients with activating genomic alterations in one of crizotinib target genes suffering from a malignancy other than its approved indication to access crizotinib. The patient commenced crizotinib 250 mg twice/day in November 2014. During the treatment, the patient had a very good performance status (ECOG 0–1), with a 100% professional activity and a stable weight. The only adverse events were grade 1 diarrhoea and asthenia, and no grade 2 or more toxicity was observed. After 3 months of crizotinib, the CT scan demonstrated stable disease, and after 15 months a partial response of − 59% in the target lesions (hepatic dome: 25 to 10 mm / left colonic flexure carcinomatosis: 36 to 15 mm). The Fig. 3 described the chronological history of the disease (A) and the centralized radiological evolution of the target lesions under crizotinib (RECIST 1.1), that concluded to 5 months partial response (B).

In March 2015, the patient noticed a progression of a left painless inguinal adenopathy of 54 mm, without progression of the other peritoneal lesions and a normal bone scan. In accordance with the protocol, a volumetric-modulated radiotherapy delivered a dose 35 Gy (10 fractions of 3.5 Gy) and crizotinib was stopped from the day before to the day after the radiotherapy. In November 2015, the periodic CT scan revealed a new node relapse (left external iliac lymph nodes of 49 mm). A CT-guided biopsy confirmed the diagnosis of papillary renal cell carcinoma, that still expressed C-MET 100% (score 3+). Therefore, due to the success of the first treatment, a 2nd radiotherapy was performed (42.5 Gy).

**Fig. 4:**
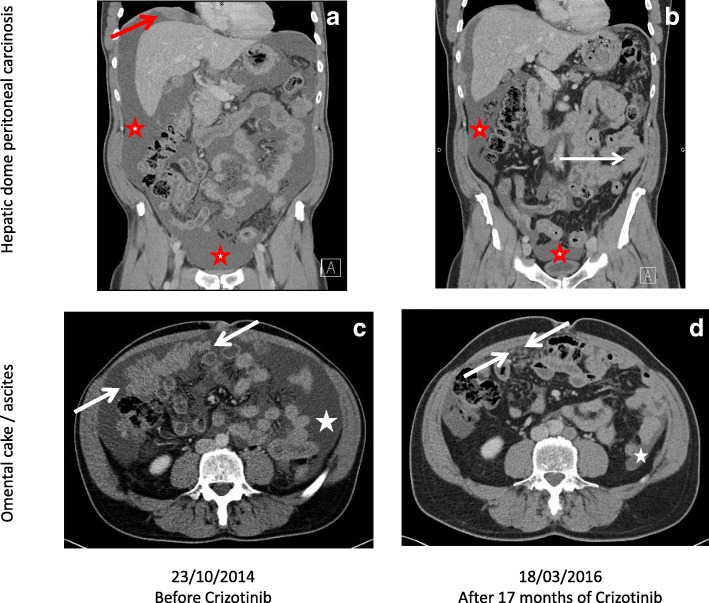
Radiological comparison of abdominopelvic CT scans before crizotininb (**a**, **c**) and after 17 months of crizotinib (**b**, **d**), in coronal (**a**, **b**) and transversal plane (**c**, **d**)

Crizotinib was continued for another 6 months, with a good tolerance (ECOG 0 with 100% professional activity) and clinical efficacy. As presented in the comparative CT scans of Fig. 4, after 17 months of crizotinib, the patient was still in ongoing response in the peritoneal carcinomatosis lesions (hepatic dome in red arrow, omental cake in white arrow) and in ascites (red and white star).

On May 2016, after 19 months of crizotinib, a relapse was observed with ascites and the patient was treated with axitinib. Three months later, the patient died of progression.

## Discussion and conclusion

MpRCC can be an aggressive disease with a lack of standard treatments, as the most classical drug in 1st line (sunitinib) was only studied in small phase II studies. In 2015, Ravaud et al. treated 61 patients with sunitinib (15 type 1 mpRCC / 46 type 2) with a modest median PFS of 6.6 months (type 1) and 5.5 months (type 2) and only 7/61 partial response [2]. Moreover, mTOR inhibitor everolimus has been shown to be less efficient than sunitinib in non-ccRCC [7, 8]. Data concerning pazopanib are scarce [9].

Here, we present the case of a man with a mpRCC, progressive after sunitinib, for which the diagnosis of *MET* amplification led to use crizotinib in 2nd line with a 19 months’ efficacy, and a very good tolerance (ECOG 0, 100% professional activity). The multidisciplinary collaboration of pathologist, molecular biologist, radiation oncologist and medical oncologist led to this successful example of personalized medicine via MET targeting. Interestingly, Diamond et al. described a pRCC patient with an activating *MET* gene mutation, pretreated by sunitinib and everolimus, with a long response to a tyrosine kinase inhibitor (TKI: PF-04217903) [10]. But this situation is rare in routine clinical practice, as *MET* copy number amplifications are more frequent in pRCC (81% of type 1 in the series of Albiges et al.) than intracellular domain *MET* mutations (21%) [11]; moreover the development of this TKI has stopped.

Biologically, the proof of *MET* implication in pRCC was made in 1997 with the discovery that hereditary pRCC patients had a germline missense mutation in the *MET* proto-oncogene (7q3 locus), leading to constitutive activation of the MET protein [12]. Recently, a translational analysis of 220 pRCC revealed the presence of DNA copy number alterations (gain) in 46% of type 2 pRCC and in 81% of type 1 pRCC, significantly higher than in clear cell RCC [11]. And it has been published earlier that gene copy number increase is associated with upregulation of MET protein expression [13]. Another large series of pRCC (*n* = 164) described *MET* mutations in 17 tumors, mainly in type 1 pRCC and in the tyrosine kinase domain (14/17), and discovered an alternate MET RNA transcript leading to a constitutive activation of MET in a ligand-independent manner [14].

This biological evidence for the implication of MET in both hereditary and sporadic pRCC have led to design trials with MET inhibitors in pRCC [15, 16]. However, the preliminary results of MET targeting were disappointing. First, foretinib (MET/VEGFR2 inhibitor) was examined in a phase II including 74 patients with mpRCC: the median PFS was 9.3 months and patients with a germline mutation were significantly more likely to respond [17]. No responses were observed in patients with *MET*-amplification (*n* = 2) and only 1/18 patients (5%) with a gain of chromosome 7 responded. Second, tivantinib (selective oral MET inhibitor) had very poor outcomes in PFS (median: 2 months) and OS (median 10 months) and had a 0% response rate (*n* = 70) [18]. Thirdly, savolitinib (AZD6094 or volitinib, selective MET inhibitor) was recently tested in a single-arm, multicenter, phase II study (*n* = 109) [19]. Interestingly, if the response rate was 0% in MET-independent pRCC, it was of 18% in patients with a MET-driven pRCC (chromosome 7 copy gain, focal MET or HGF gene amplification, or MET kinase domain mutations). To broaden this strategy of MET targeting, the SWOG 1500 (PAPMET trial, phase II) was designed as a multi-arm trial of three MET inhibitors (cabozantinib, volitinib or crizotinib) versus sunitinib [20]. This trial will include 180 patients and the first results (primary outcome: PFS) are expected for 2020. Interestingly, several biomarkers are included in the trial, either integrated (MET mutation / MET expression) or exploratory (genomic profiling), which will add precious biological knowledge.

Concerning crizotinib, this treatment is a currently available MET-ALK-ROS1 inhibitor, with a favourable toxicity profile, and is well known by medical oncologists for the treatment of non-small cell lung cancer [21, 22]. At the time we treated this patient, no data was available concerning the efficacy of crizotinib in pRCC. Only a previous case report described a MET-mutated pRCC patient pre-treated with sunitinib and tivantinib [23]; however this mutation (missense base substitution MET H1094L) is rare compared to MET amplification, and the patient only remained on crizotinib therapy for 5 months (versus 19 months in our patient). Our report also highlights the feasibility and efficacy of a focal irradiation in the situation of a pRCC patients treated with crizotininb (with suspension of crizotinib from the day before to the day after radiotherapy). Interestingly, in November 2017 were published the results of pRCC1 patients included in the CREATE trial (EORTC 90101), a multicentric prospective phase II clinical trial including patients with tumors harboring specific alterations leading to ALK and/or MET activation and treated with crizotinib [24]. Among the 4 MET-driven pRCC1 patients, the ORR was 50%, with a 1-year PFS rate = 75%, whereas among the 16 MET-independent pRCC1 patients, the ORR was 6.3% with a 1-year PFS rate = 27%. This study demonstrated that crizotinib is active in pRCC, achieving long-lasting disease control in patients with MET mutations or amplification.

Our case report illustrates and reinforces this finding that crizotinib is more active in MET driven pRCC, and that a precise molecular diagnosis is crucial for the optimal care of pRCC. This precision treatment (crizotinib) was possible thanks to an original biology-driven national French program (AcSé crizotinib NCT02034981), giving access to crizotinib for patients with identified activating genomic alterations in the crizotinib target genes, with a safe monitoring [25]. Today, this academic national program (28 regional centers), enrolled more than 235 patients with refractory malignancies and no therapeutic alternative [26]. In ASCO 2018, the results of AcSé crizotinib trials demonstrated the efficacy of crizotinib in chemo-refractory MET-amplified esogastric adenocarcinomas (ORR = 42.8%) [27]. The data are not yet mature for a specific analysis of pRCC patients, but this case report and the data of CREATE study are promising results of precision medicine in pRCC.

In conclusion, the molecular documentation of MET in pRCC seems crucial, as MET inhibitors like crizotinib are effective, safe, and nowadays available.

## Abbreviations

AcSé program: For “secure access to innovative targeted therapies”
ccRCC: Clear cell renal cell carcinoma
CEN: Centromere
ECOG: Eastern Cooperative Oncology Group
MET: Mesenchymal epithelial transition proto-oncogene
mpRCC: Metastatic papillary renal cell carcinoma
NSCLC: Non small cell lung cancer
pRCC: Papillary renal cell carcinoma
RCC: Renal cell carcinoma
RECIST: Response evaluation criteria in solid tumors
TKI: Tyrosine kinase inhibitor
